# Chronic Food Insecurity in US Families With Children

**DOI:** 10.1001/jamapediatrics.2022.5820

**Published:** 2023-02-06

**Authors:** Noura Insolera

**Affiliations:** 1 Institute for Social Research, University of Michigan, Ann Arbor

## Abstract

This survey study uses data from the Panel Study of Income Dynamics to compare trends from 2015 to 2019 in food insecurity among households with children with trends from 1999 to 2003.

In 2020, in 7.6% of all households, children were food insecure.^1^ Childhood food insecurity is associated with adverse outcomes, including anxiety, depression, poorer diet quality, higher rates of diabetes and obesity, and lower academic performance.^2,3,4^ The purpose of this survey study was to compare current trends (2015-2019) in chronic food insecurity with trends from 20 years ago (1999-2003).

## Methods

Data were obtained from the Panel Study of Income Dynamics (PSID).^5^ Balanced panels of families who participated in all 3 waves from 1999 to 2003 (n = 6029) and from 2015 to 2019 (n = 7411) were compared. Food insecurity was measured using the US Department of Agriculture (USDA) Household Food Security Survey Module (HFSSM), and categories follow the USDA Food Security Status definitions.^1^ Food security status was defined in 2 ways: (1) food insecure (low or very low food security [>2 reported HFSSM items]) and (2) not high food secure (NHFS; marginal, low, or very low food security [>0 reported HFSSM items]). Chronic food insecurity and NHFS trends were categorized by the number of waves in which these criteria were met during each 3 wave period. These mutually exclusive categories ranged from 0 (always secure or high food secure) to 3 (never food secure or high food secure). The University of Michigan institutional review board determined that approval was not needed because the data were deidentified and publicly available. All PSID participants provided oral or written informed consent. The study followed the American Association for Public Opinion Research (AAPOR) reporting guideline.

To generate nationally representative estimates and account for sample attrition, clustering, and strata, all analyses applied longitudinal family-level survey weights. Analyses were conducted using Stata, version 16 (StataCorp LLC).

## Results

**Figure 1.**
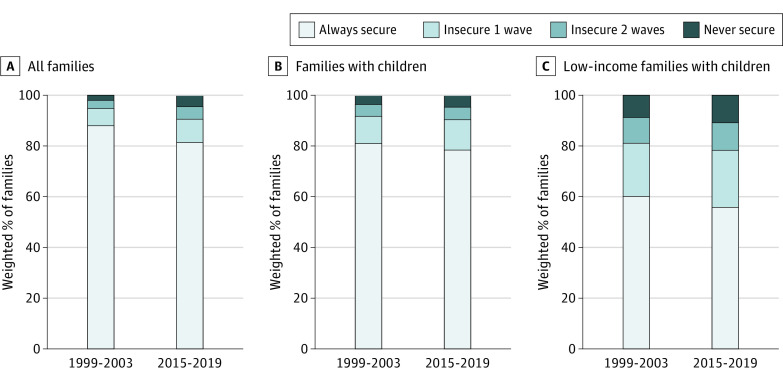
Food Insecure Occurrences From 1999 to 2003 and From 2015 to 2019 (Weighted)

Figure 1 shows that almost half of the 12.1% of families that ever reported food insecurity from 1999 to 2003 experienced at least 1 additional wave of food insecurity (5.3% of all families). From 2015 to 2019, 4.5% of all families reported food insecurity in all 3 waves, more than doubling the rate from 1999 to 2003 (2.1%). Low-income families with children experienced higher levels of chronic food insecurity compared with other families from 1999 to 2003 (8.8% of low-income families with children vs 2.1% of all families and 3.7% of all families with children). Chronic food insecurity was even higher from 2015 to 2019 (10.9% of low-income families with children vs 4.5% of all families and 4.8% of all families with children).

**Figure 2.**
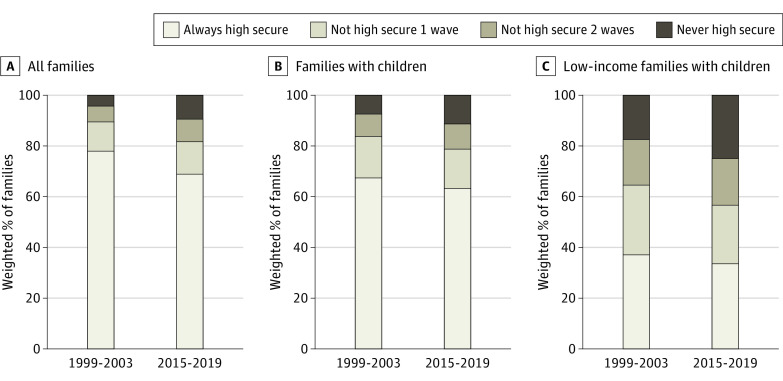
Not High Food Secure Occurrences From 1999 to 2003 and From 2015 to 2019 (Weighted)

Of the 31.2% of all families who reported NHFS status in at least 1 wave from 2015 to 2019 (Figure 2), 59.3% reported NHFS status in at least 1 additional wave (18.5% of all families). Among low-income families with children, only 33.1% always reported high food security, with 25.0% of low-income families with children never reporting high food security during the period.

Alhough the PSID has shown lower cross-sectional rates of food insecurity compared with the benchmark Current Population Survey across time, it remains the only nationally representative, longitudinal panel data on food security collected, to our knowledge.^6^ Although the HFSSM is used in both surveys, the PSID-based trends of chronic food insecurity could be underestimated.

## Discussion

The HFSSM was not collected in the PSID from 2005 to 2013, and because of this, food security status can be considered in only 2 separate 5-year periods. Also, owing to small cell sizes, analyzing low and very low food security statuses separately is not possible in this subsample.

Overall, however, these results indicate that the magnitude and dynamics of food insecurity are changing over time. Many family units cycle into and out of food insecurity over time, although a growing group remains food insecure in consecutive waves. Because of the PSID’s prospective, longitudinal collection of data, nationally representative estimates of chronic food insecurity are available for the first time in 20 years. This study begins to define these trends, with the next steps considering the mechanisms by which these changes occur.

## Notes

Supplement 1.
Data Sharing Statement
