# Supplementary material for: Chronic Food Insecurity in US Families With Children
Source: JAMA Pediatr. 2023 Feb 6;177(4):434–5. doi: 10.1001/jamapediatrics.2022.5820 (PMC10071336; doi:10.1001/jamapediatrics.2022.5820)
Supplement: Supplement 1. — Data Sharing Statement [file jamapediatr-e225820-s001.pdf]

Data Sharing Statement

Insolera. Chronic Food Insecurity in US Families With Children. *JAMA Pediatr*. Published February 06, 2023. doi:10.1001/jamapediatrics.2022.5820

Data
Data available: Yes
Data types: Deidentified participant data, Data dictionary
How to access data: https://www.openicpsr.org/openicpsr/psid
When available: With publication

Supporting Documents
Document types: None

Additional Information
Who can access the data: Anyone requesting the data who have agreed to the conditions of use.
Types of analyses: Any purpose
Mechanisms of data availability: With signed data access agreement through Open ICPSR.
